# Unilateral morning glory disc anomaly in a patient with prenatal Zika virus exposure

**DOI:** 10.1186/s40942-020-00240-1

**Published:** 2020-08-01

**Authors:** Sofía M. Muns, Mónica P. González, Victor M. Villegas, Gabriela de la Vega, Camila V. Ventura, Audina M. Berrocal

**Affiliations:** 1 Department of Ophthalmology, University of Puerto Rico, Medical Sciences Campus, San Juan, PR 00936-5067 USA; 2 Bascom Palmer Eye Institute, University of Miami Miller School of Medicine, Miami, FL 33136 USA; 3 Department of Surgery, Ponce Health Sciences University, Ponce, PR 00732-7004 USA; 4 Department of Radiology, HIMA San Pablo Hospital, Caguas, PR 00726-4980 USA; 5 Department of Ophthalmology, Altino Ventura Foundation, Recife, Brazil; 6 Department of Ophthalmology, HOPE Eye Hospital, Recife, Brazil

**Keywords:** Zika virus, Morning glory disc anomaly, Optic disc, Excavated disc anomaly, Coloboma

## Abstract

**Background:**

To report a case of morning glory disc anomaly (MGDA) in a pediatric patient with prenatal Zika virus (ZIKV) exposure.

**Case presentation:**

A 3-year-old male with prenatal exposure to ZIKV, confirmed by real-time polymerase chain reaction testing during the second trimester of pregnancy, was evaluated due to visual loss. Physical examination was remarkable for unilateral MGDA. Neuroimaging showed a base of skull encephalocele through the floor of the sella and callosal dysgenesis.

**Conclusions:**

This is the first report to suggest an association between prenatal ZIKV exposure and MGDA. Prenatal ZIKV exposure may be associated to a wider pathologic spectrum than previously reported.

## Introduction

Morning glory disc anomaly (MGDA) is a rare congenital anomaly of the optic disc and peripapillary retina [1, 2]. The prevalence of MGDA has been estimated to be approximately 2.6/100,000 [3]. Characteristic findings include an enlarged optic disc with a central funnel-shaped excavation, a hypopigmented central tuft of glial tissue, peripapillary chorioretinal atrophy, and radial spoke-like vascular pattern [1, 2, 4, 5]. MGDA is sporadic, exhibits female predominance, and is typically unilateral [1, 4, 6, 7]. MGDA has also been associated with midline craniofacial defects [1, 6]. Central visual acuity may be normal or decreased, depending on the extent of the disease [7].

The Zika virus (ZIKV) is a single-stranded RNA arbovirus that developed into a global health concern during 2016 and affected approximately 1.5 million people in South and Central America [8, 9]. The virus is mainly transmitted by the *Aedes aegypti* mosquito [8, 9]. However, vertical transmission has also been described [10]. The exposure to ZIKV during pregnancy has been associated to a wide variety of birth defects, including the congenital Zika syndrome (CZS) [10]. CZS is characterized by microcephaly, intracranial calcifications, decrease brain volume, ocular defects, congenital contractures, and hypertonia soon after birth [10, 11]. Reported ophthalmologic manifestations include congenital cataracts, congenital glaucoma, lens subluxation, iris coloboma, microphthalmia, chorioretinal atrophy, pigment mottling, optic nerve anomalies, retinal vessels attenuation, and strabismus [9, 12–18]. Intracranial and intraocular anomalies may be present symmetrically or asymmetrically.

The reported prevalence of optic nerve disease among patients in Brazil with prenatal ZKV exposure has been estimated to be 17–32% [13–17]. These abnormalities include optic disc coloboma, hypoplasia, atrophy, pallor, and increased cup-to-disc ratio [9, 10, 13–17]. In this report, we describe the clinical features associated to the first case of MGDA following prenatal ZIKV exposure.

## Case report

A 3-year-old male child with a previous medical history of strabismus and cleft lip was referred to our clinics due to an anomalous optic disc and visual loss. Perinatal history revealed that during the second trimester of pregnancy the mother developed a generalized maculopapular rash. The mother denied having any other symptoms including headache, fever, conjunctivitis, arthralgia, or myalgia. Due to her symptoms, the mother underwent a real-time polymerase chain reaction (RT-PCR) test for ZIKV with positive results. Postnatal history was remarkable for a 4 day hospitalization at the neonatal intensive care unit (NICU) due to hyperbilirubinemia treated with phototherapy. Previous ocular history was remarkable for decreased visual acuity in the right eye (OD) with sensory exotropia diagnosed at 11 months of age. Strabismus surgery had been undertaken after unsuccessful occlusion therapy with excellent anatomical outcomes.

A complete ophthalmological exam was performed. Best-corrected visual acuity was 20/200 OD and 20/20 in the left eye (OS). Intraocular pressure was normotensive in both eyes (OU). No nystagmus was present. Extraocular muscle ductions were full, but a small angle exotropia was present OD. Pupils were equally round and with an afferent pupillary defect OD. The anterior segment was unremarkable OU.

**Fig. 1:**
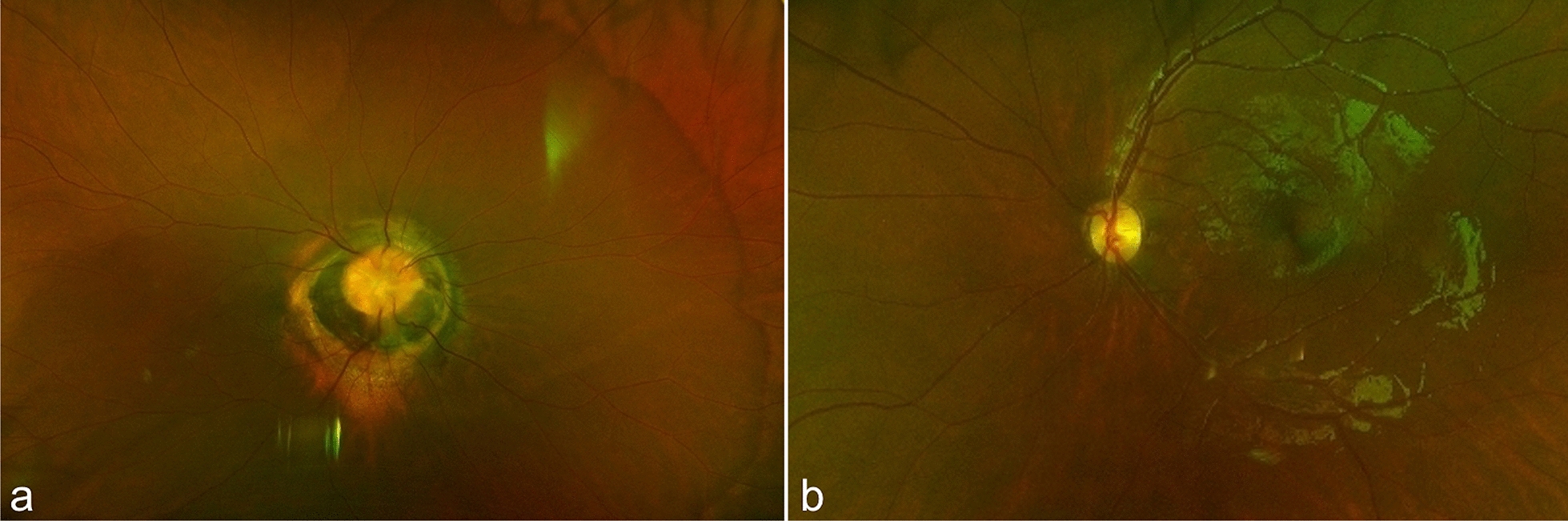
Color fundus photography of both eyes. The right optic disc (**a**) has a central funnel-shaped excavation, gliosis, peripapillary atrophy, and a radial spoke-like vascular pattern. The left optic disc (**b**) is normal

Posterior segment examination showed a centrally excavated optic disc with associated hypopigmented glial tuft, peripapillary chorioretinal atrophy, and radial spoke-like vascular pattern consistent with MGDA OD (Fig. 1). No foveal reflex was present OD. The OS posterior segment was unremarkable (Fig. 1).

**Fig. 2:**
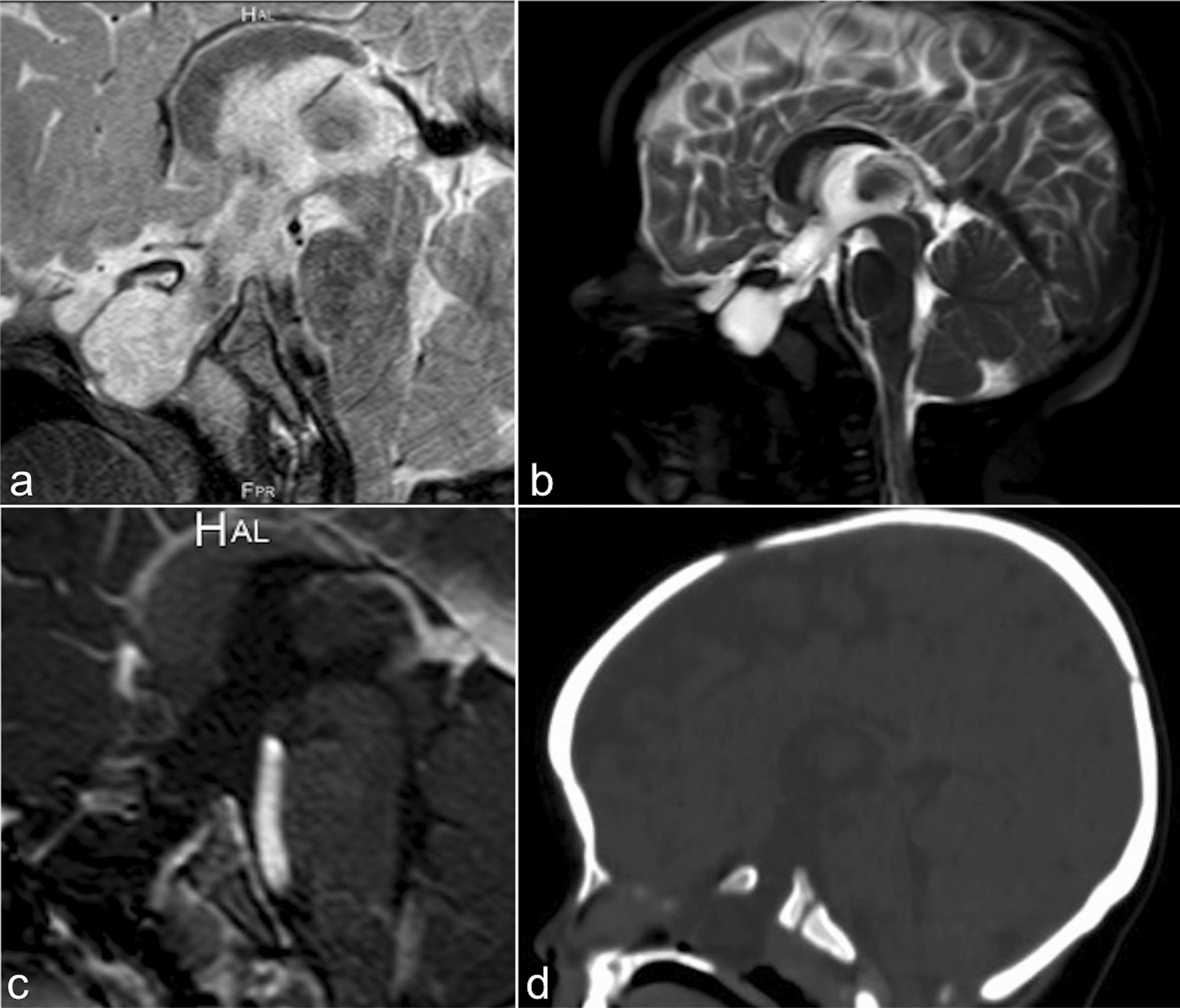
Brain magnetic resonance imaging and computerized tomography. Sagittal T2 (**a** and **b**), sagittal post-contrast T1 (**c**) weighted images, and sagittal non-contrast CT (**d**) of the brain through midline demonstrate a bony defect at the floor of the sella with herniation of cerebrospinal fluid through the defect into the sphenoid sinuses consistent with a base of skull encephalocele. There is also evidence of absence of the distal body, splenium, and rostrum of the corpus callosum consistent with callosal dysgenesis. The pituitary gland, infundibulum, and optic chiasm were not well visualized raising concern for other associated midline developmental abnormalities

Magnetic resonance imaging (MRI) and computed tomography (CT) were performed and were remarkable for a base of skull encephalocele and callosal dysgenesis. The pituitary gland, infundibulum and optic chiasm were not well visualized raising concern for other associated midline developmental abnormalities (Fig. 2).

## Discussion and conclusions

MGDA is part of a spectrum of diseases affecting the optic disc known as excavated optic disc anomalies, which also include peripapillary staphyloma, optic disc coloboma, optic disc pit, megalopapilla, and optic disc dysplasia [18]. The pathophysiological mechanisms leading to the spectrum of excavated optic disc anomalies continue to be incompletely understood [18, 19]. We report the first case of MGDA after confirmed prenatal ZIKV exposure. It is possible that ZIKV infection may alter intrauterine optic nerve development and lead to MGDA.

MGDA is a non-progressive congenital anomaly of the optic disc that has been associated with other ocular anomalies including strabismus, microphthalmia, amblyopia, leukocoria, nystagmus, eyelid hemangioma, and visual field defects [1, 2, 4–7, 19]. Complex retinal detachments and choroidal neovascularization may develop in eyes with MGDA [4, 19]. Systemic cerebral and craniofacial malformations may be present including frontonasal dysplasia, basal encephalocele, pituitary insufficiency, agenesis of the corpus callosum, hypertelorism, cleft lip, cleft palate, and cerebrovascular anomalies [1, 2, 6, 19]. Therefore, neuroimaging should be performed in all cases. In our case, neuroimaging showed a base of skull encephalocele and callosal dysgenesis. The midline brain structures including the pituitary gland, infundibulum, and optic chiasm were not well visualized.

The ZIKV may have a broad spectrum of ocular manifestations. The most commonly affected ocular structures are the retina and optic nerve [10]. Studies that have evaluated infants with prenatal ZIKV exposure have reported optic disc anomalies in 17–32% of subjects [13–17]. The spectrum of optic disc anomalies reported included optic nerve hypoplasia, optic disc coloboma, increased cup-to-disc ratio, and optic nerve atrophy [9, 10, 13–17]. Although bilateral optic disc involvement is most commonly found, unilateral optic disc findings have been reported in up to 30% of cases [13–17]. Additionally, while ocular involvement most commonly occurs among patients with microcephaly, previous studies have described optic disc anomalies among patients without microcephaly [14–17]. Interestingly, no reports of MGDA in children with prenatal ZIKV exposure have been documented.

Van den Pol and associates [20] have theorized that after ZIKV is vertically transmitted, it may invade the cortical progenitor cells inside the fetal brain. The virus may then infect, via axonal transport, other parts of the visual system including the retina, optic chiasm, suprachiasmatic nucleus, and superior colliculus [9, 20]. Some histologic studies have suggested that glial cells within the optic chiasm and optic tract may be an important target of the ZIKV [9, 20]. To support this hypothesis, Fernandez et al. performed a histopathological evaluation of four deceased fetuses and isolated ZIKV from the optic nerve, neural retina, and choroid [21]. The intracranial anomalies that have been associated to MGDA overlap with those reported in some cases of CZS. It is possible that early glial cell dysfunction inside the optic nerve may lead to MGDA. Future studies may determine the exact mechanisms that lead to MGDA.

To our knowledge, this is the first study to report a case of MGDA in a patient with prenatal ZIKV exposure. The rarity MGDA may explain the lack of previous reports among children with prenatal ZIKV exposure [22, 23]. Although our report cannot demonstrate a causal relationship between prenatal ZIKV exposure and MGDA, their association should be explored in future research studies. This report suggests that prenatal exposure to ZIKV may lead to a wider pathological spectrum than previously described.

## Data Availability

All data generated or analyzed during this study are included in this manuscript.

## Abbreviations

ZIKV: Zika virus
CT: Computerized tomography
MRI: Magnetic resonance imaging
MGDA: Morning glory disc anomaly
RT-PCR: Real time polymerase chain reaction
OD: Right eye
OS: Left eye
OU: Both eyes
CZS: Congenital Zika syndrome
